# Impact of a Gender-Neutral HPV Vaccination Program in Men Who Have Sex with Men (MSM)

**DOI:** 10.3390/ijerph18030963

**Published:** 2021-01-22

**Authors:** Javier Díez-Domingo, Víctor Sánchez-Alonso, Rafael-J. Villanueva, Luis Acedo, José Tuells

**Affiliations:** 1 Vaccine Research Area, FISABIO-Public Health, Avda. Cataluña, 21, 46020 Valencia, Spain; javier.diez@fisabio.es; 2 Instituto Universitario de Matemática Multidisciplinar, 8G building, 2nd Floor, Camino de Vera, Universitat Politècnica de Valéncia, 46022 Valencia, Spain; vicsana3@doctor.upv.es (V.S.-A.); rjvillan@imm.upv.es (R.-J.V.); 3 Department Mathematics, University of Extremadura (Centro Universitario de Plasencia), 10600 Plasencia, Spain; acedo@unex.es; 4 Department of Community Nursing, Preventive Medicine and Public Health and History of Science, University of Alicante, San Vicente del Raspeig, 03690 Alicante, Spain

**Keywords:** human papillomavirus, network model, vaccination strategies, men who have sex with men, sexual behavior, sexual partners, epidemiologic transition

## Abstract

A major challenge in human papillomavirus (HPV) vaccine programs is the universal gender-neutral recommendation, as well as estimation of its long-term effect. The objective of this study is to predict the added benefit of male vaccination, especially in men who have sex with men (MSM), and to analyze the impact of the program on society. We propose a mathematical model of the HPV infection based on a network paradigm. Data from Spain allowed constructing the sexual network. HPV force of infection was taken from literature. Different scenarios using variable vaccine coverage in both males and females were studied. Strong herd immunity is shown in the heterosexual population, with an important decrease of HPV 6/11 infections both in men and in unvaccinated women with an only-women vaccination at 14 years of age. No impact of this program occurred in the infection incidence in MSM. This group would only benefit from a vaccination program that includes males. However, the impact at short term would be lower than in heterosexual men. The protection of MSM can only be achieved by direct vaccination of males. This may have important consequences for public health.

## 1. Introduction

Disparities in cancer burden between different populations, including differences associated with sexual orientation are well known. Many agencies work to identify and address the factors that lead to health inequalities [1]. Regarding cancers linked to human papilloma virus (HPV) infection, the World Health Organization’s Director considers cervical cancer as a public health problem in which the HPV vaccine is a cornerstone in their recommendation for prevention and control [2].

The HPV infection also increases the risk of other cancers as anogenital tract and oral cavity in both males and females [3,4] and could increase the risk of cancer among partners of patients with HPV-related cancer [5]. However, most countries who have developed HPV vaccination programs target girls only, based on mathematical modeling and predictions [6].

The coverage with ≥1 dose of HPV vaccine, and the percentage of adolescents who were up to date with the HPV vaccination series increased in 2019 and both measures improved among females and males [7,8,9], but there was no significant increase in either of those measures when analyzing the male only subgroup in Netherland [10]. In countries with high HPV vaccine coverage, there is a large reduction of genital warts in non-vaccinated males [11,12,13], that was predicted also in mathematical models.

There is therefore a marginal benefit of vaccinating males and this practice might not be cost-effective [14,15,16]. However, the lack of gender neutrality in this vaccine programs, supposes a concerning inequity, especially for men who have sex with men (MSM) who are at greatly increased risk of HPV associated anal cancer [17,18]. In this way, not only are HIV-positive MSM at even greater risk [17,18,19,20], but also their partners, people living with HIV/AIDS (PLWHA) [21,22].

Recent reports have noted increasing rates of anal and oral cavity and pharynx cancer among high-income countries worldwide [23,24,25,26,27] all HPV-related. Chatuvedi AK, et al. [25] in 2011 showed a rising at 5% annually of incidence of all oropharyngeal cancers and has surpassed that of cervical cancer in women for the first time in 2010 and the annual number of cases in the USA is projected to almost double by the year 2030 [25]. In the United States (US), the five-year relative survival of oropharyngeal cancer patients increased from 33.3% in 1992–1996 to 42.2% in 2002–2006 [28].

The effectiveness of HPV vaccination programs can be affected by different factors related with the sexual behavior, age difference between sexual partners [29].

A vaccination program targeting MSM may have low benefit as most of them would have been infected already at the time of vaccination [30], when the vaccine is less effective. Moreover, reaching this population has been insufficient in other programs, as occurred in Spain with the HepA vaccine during a disease outbreak in MSM and there is no reason to suspect the same would not apply to this vaccination program [31].

During the past decade, many models have been developed to understand HPV infection epidemiology and to help guide policy decisions regarding vaccination [6] and are quite consistent among different models [6,32]. However, these models vary in terms of type (deterministic or stochastic), structure (assumptions about sexual activity, partnership formation and dissolution, transmission, and natural immunity), and baseline HPV prevalence. The modelling of Qendriet et al. [33] supports that sex-neutral vaccination can be used to bolster herd immunity to women and to directly protect men, particularly MSM, with the clinical significance of either argument determined by the achieved coverage. 

The objective of the study is to estimate by lifetime sexual partners (LSP) network mathematical modeling, the impact of a gender-neutral vaccination program on the HPV infection, focusing on the impact on MSM, with current vaccine coverage in Spain (about 70% in adolescents).

## 2. Materials and Methods

### 2.1. Computational Network Model Building

The sexual network consists of nodes (sites representing the individuals) and links or bonds between partners representing contacts where the infection may spread. Using this network, it is possible to simulate the evolution of transmission of an infectious disease over time. In network models, we can follow any individual and select those targets for a vaccination program, which is more difficult in continuous models.

For the construction of the sexual network, as there is no complete information on the sexual behaviors in a society, we assumed that a large-scale network of sexual contacts would be similar to a random network, where individuals or node bonds are assigned at random, but taking into account some similarities among individuals related to age and promiscuity. Data on sexual behavior specific from Spain were collected from the Health and Sexual Habits Survey 2003 [34].

Table 1 indicates the proportion of individuals with a given number of LSP from 0 to 10 or more, for each sex and age group. The LSP refers to the total number of sexual partners of an individual until the moment in which the survey was conducted. An algorithm was developed to match sexual partners that satisfy the conditions given in Table 1 [35,36].

MSM population represent 3.88% of the total male population in Spain [34] and were included in the model as a sub-network. These had a larger number of LSP than the heterosexual men (39 in MSM, 8 in heterosexual men) [37] and some had also random contacts with women that bridge between the two networks.

This model takes into account infections by the most common genotypes in Spain and has been calibrated and simulated for HPV genotypes that cause cancer and genotypes that cause genital warts (HPV 6/11), all covered by nine-valent vaccine.

Infections are simulated with a susceptible-infectious-susceptible (SIS) model in which every node/individual may be susceptible or infected by HPV.

A set of probabilities were considered and calibrated to explain the transitions among SIS states:

- The global frequencies of the intercourses per age group and time step.
- The average recovery time from HPV 6/11 infection.
- Two parameters determining if the HPV 6/11 infection is transmitted from a man or woman to his/her partner (force of infection).

To calibrate the model, we used data from the CLEOPATRE study [38] in which the prevalence of different HPV genotypes in women was determined for a selected population in Spain. This calibration has been performed probabilistically, returning 95% confidence intervals of the model parameter values and the model outputs [36].

The network consisted of 100,000 nodes, and the vaccine effectiveness was assumed to be 96.5% in naïve subjects of both genders, and between 20% and 54% in MSM when requiring vaccination [39].

### 2.2. Scenarios to Be Simulated

The population modelled were all persons 14 to 64 years of age, so when each member of the community enters the model, they were already vaccinated depending on the vaccine coverage. The temporal length of the model was 100 years, and each temporal step corresponded to one calendar month.

We assessed different scenarios:

- Scenario 1: Only girls were vaccinated with a coverage of 70% (current vaccination program in Spain).
- Scenario 2: 70% of both girls and boys were vaccinated.
- Scenario 3: 70% of girls were vaccinated and 20% of MSM 18 to 45 years starting 15 years after the girls’ program (to resemble most of the programs).

We calculated the decline of the prevalence of HPV infection on women, overall men and MSM over time measured as

D_t_ = 100 (1 − P_t_/P )

where D_t_ is the decline of the HPV infections in the time instant t, P_t_ is the prevalence of the HPV infection in the time instant *t* and P is the prevalence of the HPV infection with no vaccination program.

To estimate the percentage of HPV infections averted in both scenarios, we compared the prevalence of infection at a moment with a non-vaccination scenario and use the formula

A_t_ = 100 (1 − P_t_/N_t_ )

where A_t_ is the percentage of HPV infections averted in the time instant t, P_t_ is the prevalence of the HPV infection in the time instant *t* in scenario 1 or 2 and N_t_ is the prevalence of the HPV infection in the non-vaccination scenario.

Taking into account the short period of time in developing genital warts when infected by HPV 6/11 (6 months), we can assume that HPV 6/11 decline (percentage of averted cases) is similar to decline (percentage of averted cases) in genital warts.

### 2.3. Sensitivity Analysis

As sexual behaviors are continuously changing; we increased the percentage of MSM to 10%. That implied an increase in the number of LSPs of MSM around 160% compared to the case base.

The effectiveness of the vaccine when used in MSM was considered between 20 and 54%, therefore two models of maximum and minimum effectiveness were described.

To avoid the inconveniences of having data on sexual behavior specific from Spain of 2003, we have published a model sensitivity analysis, we found that large changes in the sexual behavior, in some extent, only have minor effects on the decline of the HPV infections in women and men in the current vaccination campaign in Spain (vaccination of young girls with a coverage of 70%) [40].

## 3. Results

Network mathematical models is a path forward in the modeling of the epidemiology of infectious diseases. We used this model to predict the epidemiology of respiratory syncytial virus (RSV) in babies and toddlers [41], and with this sexual network we maneuver to incorporate LSP.

**Figure 1:**
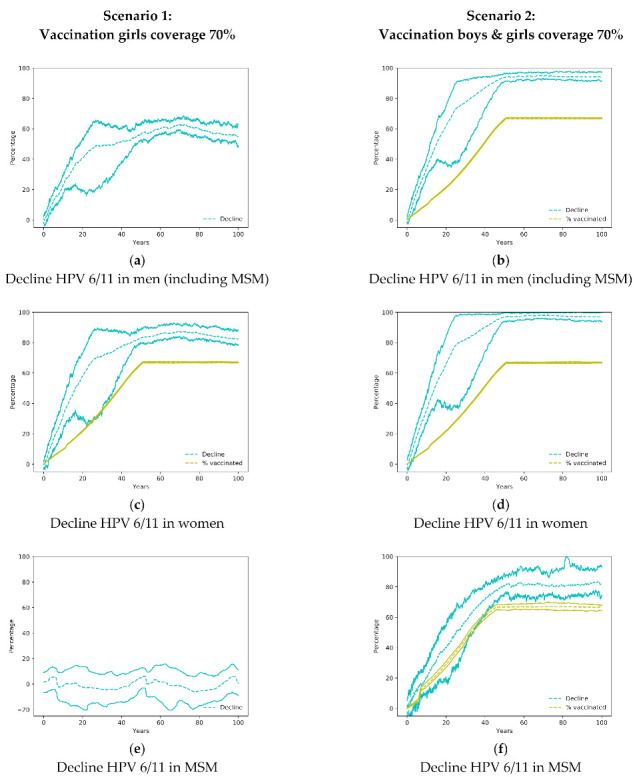
Decline of HPV 6/11 in men, MSM and women in the simulated scenarios 1 and 2. In the figures where a yellow line appears denoting the percentage of vaccinated, the difference between the yellow line and the blue lines is a measure of the community effect. MSM: men who have sex with men; HPV: Human Papilloma Virus.

The sexual network model here presented has already been used to study the epidemiology of HPV infection before and after vaccination and reflected the fast decline of genital warts in Australia after the immunization campaign [35,36]. A vaccine coverage of 70% is reached in the whole studied population at 50 years, i.e., when the first vaccinated cohort reaches 65 years old and then stabilizes [35]. The vaccination coverage and the reduction of HPV infection in the whole cohort 14–64 years old in different populations and scenarios are shown in Figure 1.

### 3.1. Community Effect in the Heterosexual Population

Shortly after the implementation of the only girls’ vaccination program, there is a large impact on the transmission of HPV 6/11 genotypes with a reduction of HPV infections in both women and men (Figure 1a,c). That initial impact is due to a large number of sexual encounters and a high probability of infections in youngsters. The only girls’ vaccination program has also an important effect on males so, in the first 20 years of the vaccination program, the decline of infections in males is larger than the vaccination coverage in women (Figure 1a,c). When both, males and females are vaccinated (Scenario 2), the reduction of infections is larger and sharper in both sexes (Figure 1b,d).

### 3.2. Impact on Men Who Have Sex with Men

The vaccination of women only (Scenario 1) does not affect HPV infection amongst MSM (Figure 1c). Only when boys have been vaccinated does the reduction of infections in MSM occur but at a lower rate than heterosexual men, as the herd immunity is reduced due to lesser impact of the vaccination of females.

**Figure 2:**
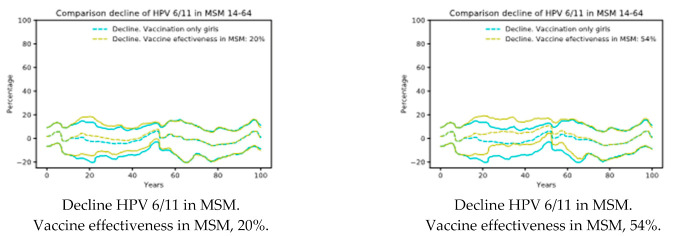
Scenario 3: Vaccination girls’ coverage 70% and 20% of MSM 18 to 45 years vaccinated. Decline of HPV 6/11 in MSM depending on the vaccine effectiveness [39]. Note that there are small effects in the decline. MSM: men who have sex with men.

Targeted vaccination programs do not seem to be effective. In Figure 2, we present the results of scenario 3, having considered effectiveness ranging from 20–54% in MSM. As shown in Figure 2, the results did not differ from female only vaccination practice (Scenario 1).

### 3.3. Averted HPV 6/11 Infections

Results about the percentage of averted HPV 6/11 infections are shown in Table 2. In Scenario 2 the effect of the vaccine on the MSM population proves its efficacy. Men (heterosexual and MSM) also show an enhanced protection, improving the percentage of adverted infections by almost a factor of two, approximately, in the next fifty years after the beginning of the simulated vaccination campaign.

Table 2 shows the percentage of the average averted cases in Spain comparing scenarios 1 and 2 with the scenario of no vaccination, since the beginning of the vaccination campaign after 10, 20, 30, 40, and 50 years. MSM were also included in the Men group. The differences between the percentage of vaccinated individuals and the percentage of averted cases serves as a measure of the community effect of the vaccine (herd immunity).

### 3.4. Sensitivity Analysis

We propose the following scenarios to be simulated in order to perform a sensitivity analysis:

- Case base. This is the current situation, with the percentage of MSM equal to 3.88%, and the current program to vaccinate young girls with a coverage of 70%.
- Case 1. It is the case base increasing the percentage of MSM up to 10%.

The increase of MSM up to 10% implies an increase in the number of LSPs of MSM around 160%, compared to the case base.

**Figure 3:**
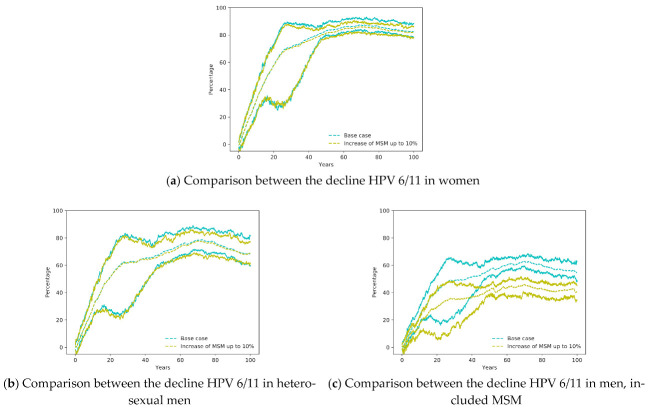
Comparison between the Base Case (vaccination of young girls with coverage 70% and 3.88% of MSM) with the same scenario with an increase of MSM up to 10%.

Figure 3a,b show very similar declines in both scenarios for women and heterosexual men. Therefore, the increase of MSM does not affect significantly the decline of HPV infections in women. However, there is a greater difference in the decline of HPV infection in the men group, which can be attributed to a diminished decline in the MSM group since heterosexual men does not include them and showed almost no difference).

## 4. Discussion

Prophylactic HPV vaccine programs constitute major public health initiatives worldwide and modelling studies have been and still are widely used to inform HPV vaccination policy decisions [6], the analysis we have undertaken enables an assessment of the impact that a vaccination program targeting a minority group, the MSM population, that suffers an increased risk of HPV infections and its associated cancers, could reach. These kind of assessments are required in order to visualize the benefits of any new health intervention.

Using LSP network models [35] could have a benefit over the classical continuous models, defining more specifically the transmission of the infection, and reflecting precisely the herd immunity. In addition, the LSP network models consider the structure of sexual contacts and reflects the protection of the vaccine in infection control. In addition, it is more restrictive for the spread of the HPV than the usual assumption in the continuous models where anybody can infect anybody (homogeneous mixing) [42].

Our findings are in agreement with those found by Brisson, et al. [6], on the important effects of vaccination only for girls, for both men and women, but our analysis, focused on the MSM population, shows that girls-only vaccination has close to no effects on this population and that a gender-neutral immunization programme has an adequate impact on MSM.

In this way, our study is also consistent with the modeling carried out by Bogaards J. et al. [43] for MSM in the Netherlands, who explored the potential effectiveness of HPV vaccination for MSM based on models of penile-anal transmission of HPV16. They found a reduction in the prevalence of HPV16 among MSM exceeding vaccine coverage projections, illustrating the efficiency of prophylactic immunization even when the HPV vaccine is administered after sexual initiation [43]. In the same way, the modeling of Gao, S. et al. [44] found that the heterosexual population gets great benefit, but MSM only get minor benefit from vaccinating heterosexual females or males.

Our model suggest that elimination of HPV 6/11, is likely if vaccination coverage of girls and boys reaches 70% and the vaccine provides long-term protection.

On the other hand, we modeled a scenario with a target vaccination program for MSM. We found no changes in the evolution of HPV infection in this group. There were no changes in the decline of the infection even after increasing the vaccination coverage, in contrast to Gao S. et al. [44], who suggests the best vaccination strategy is to vaccinate MSM. Bogaards J. et al. [43] also suggested that HPV vaccination might be effective when given to MSM. The inclusion of older MSM would likely be needed to achieve substantial vaccine coverage and impact. However, our model predicts that a targeted program will not produce changes in the course of HPV infection, even if the effectiveness reached 54% in MSM. All these discrepancies could be relative to the type of modeling than to the modeling population.

Information on sexual orientation disparities in cancer incidence and mortality is not available for the general population, and in general is mostly inferred from populations with HIV/AIDS [1], mainly due to difficulties in collecting data about sexual orientation. Additionally, health care systems have difficulties to correctly care for people with social and cultural customs differing from the baseline population. In order to achieve a prolonged and consistent impact preventing HPV infection in MSM a gender-neutral vaccination program is necessary, as it is the only strategy that offers MSM a level of protection similar to the other compared groups.

In this study, we showed that having a universal vaccination program for both girls and boys will have an important effect on the epidemiology of HPV cancers. Moreover, even with a persistent 70% vaccine coverage in the current vaccination programs, the infection will persist in the MSM population as current models do not achieve correct protection of this subgroup of the population.

In this work we have analyzed the community effect for HPV 6/11 vaccination in Spain. Also, other scenarios have been analyzed in order to assess the impact of a gender neutral vaccination program on MSM.

To achieve this objective, we have applied a recent model for the network of sexual contacts in a population that can be calibrated by modifying the number of lifetime sexual partners according to new evidence or to create new simulations. This mathematical model based upon the network paradigm has been successfully used to study the stable number of HPV infections [36] as well as the fast decline in the number of genital wart cases in Australia [35].

In the particular case of Spain, we have found in the near future the community effect will be very evident for the male population, with a decline in the percentage of cases, approximately, the double of the percentage of cases directly prevented in females as a consequence of vaccination. The results in the female population show a more likely improvement, but at the very least the current levels of protection will be sustained.

This data corresponds with a simulation on the population of Spain for a sustained vaccination campaign keeping a coverage around 70% for girls aged 14 years old. Our study has also spotted an important handicap of the present vaccination program that implies low or null community effect in the MSM population. One of the possible causes for this result may be due to the greater number of sexual partners, in which part of this group may have events with women, as has also been described for this group of the population in other countries [45].

This may have important consequences for public health policies as it is a question under discussion at the moment the inclusion in the vaccination campaigns. To evaluate this second scenario, we have studied the effect of 70% coverage in both boys and girls to analyze the number of averted cases of genital warts directly caused by HPV 6/11 infections. The improvement for heterosexual men is more or less doubled in the following decades with a protection of MSM as well. For these reasons, the recommendation of boys’ vaccination with the HPV vaccine is supported by these results.

### Limitations of the Model

There are many possible combinations to create networks fulfilling the requirements in Table 1. This fact makes that the built networks contain uncertainty in the sense of the randomness of the building process and the different shapes these networks may have.

The computational network model developed depends on the data used in its construction. Thus, it will be influenced by possible biases in the data about sexual behavior [26] and the source data to calibrate it [38]. To check that the possible bias is low, we have performed a sensitivity analysis published in [40].

The lack of specific data about sexual behavior of the population do not allow us to check the reliability of the network structure. However, the computational network model built reproduces accurately real situations [35].

Our analysis did not include scenarios with naturally acquired immunity or reactivation of latent infections since the mechanisms of immunity and latency are not well understood yet.

## 5. Conclusions

The sexual network model built predicted that the vaccination of only women leaves MSM without protection and informs that their protection can only be achieved by the direct vaccination of males. This may have important consequences on public health.

## Figures and Tables

**Table 1:** Proportion of males and females per number of lifetime sexual partners per age group.

**Males**						
**Age**	**0 LSP**	**1 LSP**	**2 LSP**	**3–4 LSP**	**5–9 LSP**	**10 or more LSP**
14–29	0.107	0.207	0.131	0.225	0.168	0.162
30–39	0.027	0.225	0.128	0.21	0.17	0.24
40–65	0.019	0.268	0.14	0.193	0.163	0.217
**Females**						
**Age**	**0 LSP**	**1 LSP**	**2 LSP**	**3–4 LSP**	**5–9 LSP**	**10 or more LSP**
14–29	0.138	0.43	0.186	0.158	0.056	0.032
30–39	0.029	0.501	0.168	0.177	0.077	0.048
40–65	0.017	0.652	0.138	0.118	0.039	0.036

LSP: lifetime sexual partners.

**Table 2:** Averted HPV 6/11 infections.

			Years		
	10	20	30	40	50
Men * (Scenario 1)	9%	26%	41%	48%	55%
Men * (Scenario 2)	16%	49%	73%	86%	93%
MSM (Scenario 2)	10%	38%	57%	72%	79%
Women (Scenario 1)	14%	45%	66%	75%	82%
Women (Scenario 2)	18%	54%	80%	89%	97%
% of vaccinated women (both scenarios)	9.5%	21.6%	35.3%	51%	65.7%
% of vaccinated men (Scenario 2)	10%	21%	34.6%	51%	65.7%

MSM: men who have sex with men; * Include MSM.

## Data Availability

Data is contained within the article or easily accessible through the references.
